# Comparison of Antimicrobial Consumption Patterns in the Swiss and Danish Cattle and Swine Production (2007–2013)

**DOI:** 10.3389/fvets.2017.00026

**Published:** 2017-03-02

**Authors:** Luís P. Carmo, Liza R. Nielsen, Lis Alban, Cedric R. Müntener, Gertraud Schüpbach-Regula, Ioannis Magouras

**Affiliations:** ^1^Vetsuisse, Veterinary Public Health Institute, University of Bern, Bern, Switzerland; ^2^Faculty of Health and Medical Sciences, Department of Veterinary and Animal Sciences, University of Copenhagen, Copenhagen, Denmark; ^3^Danish Agriculture & Food Council, Copenhagen, Denmark; ^4^Institut für Veterinärpharmakologie und -toxikologie, Vetsuisse, University of Zurich, Zurich, Switzerland

**Keywords:** antimicrobial consumption, antibiotics, antimicrobial resistance, Switzerland, Denmark, pigs, cattle

## Abstract

Veterinary antimicrobial consumption patterns vary considerably across Europe. These differences are not only limited to the total amount consumed but are also observed with regards to the relative proportion of the various antimicrobial classes used. Currently, most of the data on veterinary antimicrobials are reported at sales level without any information on the consumption by different animal species. This hinders a proper comparison of antimicrobial consumption at the species level between countries. However, it is imperative to improve our understanding on antimicrobial usage patterns at the species level, as well as on the drivers contributing to those differences. This will allow for development of tailored interventions with the lowest possible risk for human health, while ensuring effective treatment of diseased livestock. An important step to attain such an objective is to perform detailed comparisons of the antimicrobial consumption in each species between countries. We compared antimicrobial consumption estimates for cattle and pigs in Switzerland and Denmark, in order to distinguish species-specific patterns and trends in consumption from 2007 to 2013. Swiss data were obtained from a previous study that assessed methodologies to stratify antimicrobial sales per species; Danish antimicrobial consumption estimates were assembled from Danish Integrated Antimicrobial Resistance Monitoring and Research Programme reports. A decrease in antimicrobial consumption in milligrams per kilogram of biomass was observed for both countries (4.5% in Denmark and 34.7% in Switzerland) when comparing 2013 to 2007. For pigs and cattle, the overall consumption per kilogram of biomass of most antimicrobial classes was higher in Switzerland than in Denmark. Large variations in the relative consumption of different antimicrobial classes were also evident. Sulfonamides/trimethoprim and tetracyclines were consumed in a higher proportion in Switzerland than in Denmark, whereas the relative consumption of penicillins was higher in Denmark. The differences observed in veterinary antimicrobial consumption are not solely related to animal demographic characteristics in these two countries. Other factors, such as the level of biosecurity and farming practices, veterinarians and farmers’ education, or governmental/industry programs put in place might also partly explain these variations. These differences should be taken into account when aiming to implement targeted interventions to reduce antimicrobial consumption.

## Introduction

Considerable differences in veterinary antimicrobial sales have been observed across European countries. This heterogeneity is not only evident in the total amounts but also in the proportions of the different antimicrobial classes sold (1–5).

A variety of factors might be related to the abovementioned variations: infection status (at farm and country level), animal husbandry practices and general animal keeping conditions, on-farm biosecurity and targeted disease control programs, prescription practices, product availability and price, veterinarians’ and farmers’ preferences over specific products, as well as farmers’ and veterinarians’ education and habits (6–9).

The reports from the European Surveillance on Antimicrobial Consumption (ESVAC) project provide an initial basis for comparing antimicrobial sales between European countries (1–5). However, they do not include data on consumption at the species level, but only sales data normalized to biomass of food-producing animals. When assessing overall sales data, it should be noted that animal demographics are heterogeneous among European countries. This could partly explain some of the variations observed in the total antimicrobial sales (6). Furthermore, the level of antimicrobial exposure for individual animals varies greatly between production sectors (10). With that in mind, more meaningful comparisons can be achieved by analyzing antimicrobial use at species level. In addition, overall sales do not allow the implementation of benchmarking strategies, thus targeted approaches to reduce antimicrobial consumption are not feasible.

Nonetheless, only few countries collect data at the species level. In Switzerland, sales data have been collected from marketing authorization holders since 2004 (11). No national data are available at farm level, but a methodology has recently been developed to attribute sales data to animal species (12). Denmark has recorded antimicrobial prescription data since 2001 in the VetStat database (13). The comparison between these two countries is of particular interest as, despite similar animal health status, Switzerland is characterized by a higher antimicrobial consumption in livestock per kilogram of animal biomass compared to Denmark (5). This indicates that additional factors, beyond the animal health status, might drive antimicrobial consumption in both countries. A first step to a profound understanding of these differences is to investigate which species and antimicrobial class combinations differ most between the two countries. Benchmarking antimicrobial consumption (at the species level) between countries can improve the value of these data sources and can lead to more tailored and effective reduction measures. If differences in the overall and relative consumption of different antimicrobial classes exist at the species level, there is a need to highlight them and study potential underlying factors, in order to develop more specific interventions. In addition, the successful implementation of interventions in one country could provide evidence-based motivation for other countries to follow their paradigm.

We analyzed available antimicrobial consumption data from Denmark and Switzerland with the following objectives: (a) to compare the patterns and trends of antimicrobial consumption in cattle and pigs and (b) to compare the relative consumption of different antimicrobial classes in the different species and countries.

## Materials and Methods

### Antimicrobial Consumption Estimates in Switzerland

In a previous study, Swiss sales of all veterinary antimicrobials products were stratified by animal species (12). Consumption estimates for cattle and pigs referred to in the present study were derived from the model entitled “Longitudinal Study Extrapolation” (LSE). In the LSE approach, sales data were combined with information from a field study by Regula et al. (14), where prescription patterns of 8 veterinary practices (with a total of 15 veterinarians) were investigated. The veterinary clinics enrolled in the study were selected based on the proportion of owners keeping livestock and the availability of electronic databases for disease and prescription records; they represented 1.5% of all large and mixed veterinary clinics in Switzerland. The animal species included in this study were cattle, pigs, sheep, goats, horses, dogs, and cats (14). To take uncertainty into account, Pert distributions were used in Monte Carlo simulation models to estimate the total amount of antimicrobials consumed by each animal species in a year and at antimicrobial class level. Prescription practices from the study by Regula et al. (14) were used to estimate the mode of the Pert distributions. Minimum and maximum were calculated using official sales data by the Swiss Federal Food Safety and Veterinary Office by taking into account which products were licensed for each species.

These results represent a proxy of antimicrobial consumption. To improve readability of the manuscript, estimated consumption will be referred to hereafter as “consumption.”

### Antimicrobial Consumption Estimates in Denmark

Denmark developed an electronic database (VetStat) to collect veterinary prescription information in 2001 (13). Data from all antimicrobial prescriptions at species level are published annually in the reports from The Danish Integrated Antimicrobial Resistance Monitoring and Research Programme (DANMAP). Data on cattle and pig’s consumption (2007–2013) of different antimicrobial classes were collated from the DANMAP reports (15–21).

Between 2007 and 2009, antimicrobial consumption data were reported in DANMAP as prescriptions made directly to the farmers and antimicrobials sold for use in veterinary practice. For this time period, these values were added up to estimate the total antimicrobial consumption for each species. From 2009 onward, prescriptions made directly to the farmers and antimicrobials sold for use in veterinary practice were already summed up when presented in DANMAP reports.

### Animal Demographic Data

The national total biomass was calculated for each animal species according to the population correction unit (PCU) method (1). Average animal weights followed the ESVAC recommendations. Animal population data for each study year were obtained from ESVAC reports (1–5). Cattle data included all production bovines in the countries—dairy and beef cattle. It should be noted that dairy and non-dairy proportions varied between the two countries (22, 23).

### Comparison between the Two Countries

Antimicrobial consumption was calculated in milligrams per kilogram biomass (mg/BM) annually from 2007 to 2013. This metric differs from milligrams per PCU solely by the fact that the latter was coined to specifically account for the biomass of multiple animal species in the denominator, while mg/BM can be calculated for specific animal species.

Unless stated otherwise, differences on antimicrobial consumption over the study period were calculated by subtracting the antimicrobial consumption in mg/BM (or corresponding percentage) in 2013 and 2007.

Specific antimicrobial class consumption estimates were also calculated for each species and country. The relative consumption of each antimicrobial class in each country was calculated as

$${\text{Relative consumption}}_{\text{antimicrobial class, species, year}}=\frac{\text{mg}/{\text{BM}}_{\text{antimicrobial class, species, year}}}{\text{mg}/{\text{BM}}_{\text{species, year}}}\times 100$$

### Data Analysis

Data management and descriptive analyses were performed in MS Excel (24). A correlation analysis between years and antimicrobial consumption was performed for each combination of species/antimicrobial class/country. The Pearson correlation coefficient was used as measure. This analysis was done using the statistical software R version 3.1.0.

## Results

### Antimicrobial Consumption Estimates in Cattle

**Figure 1:**
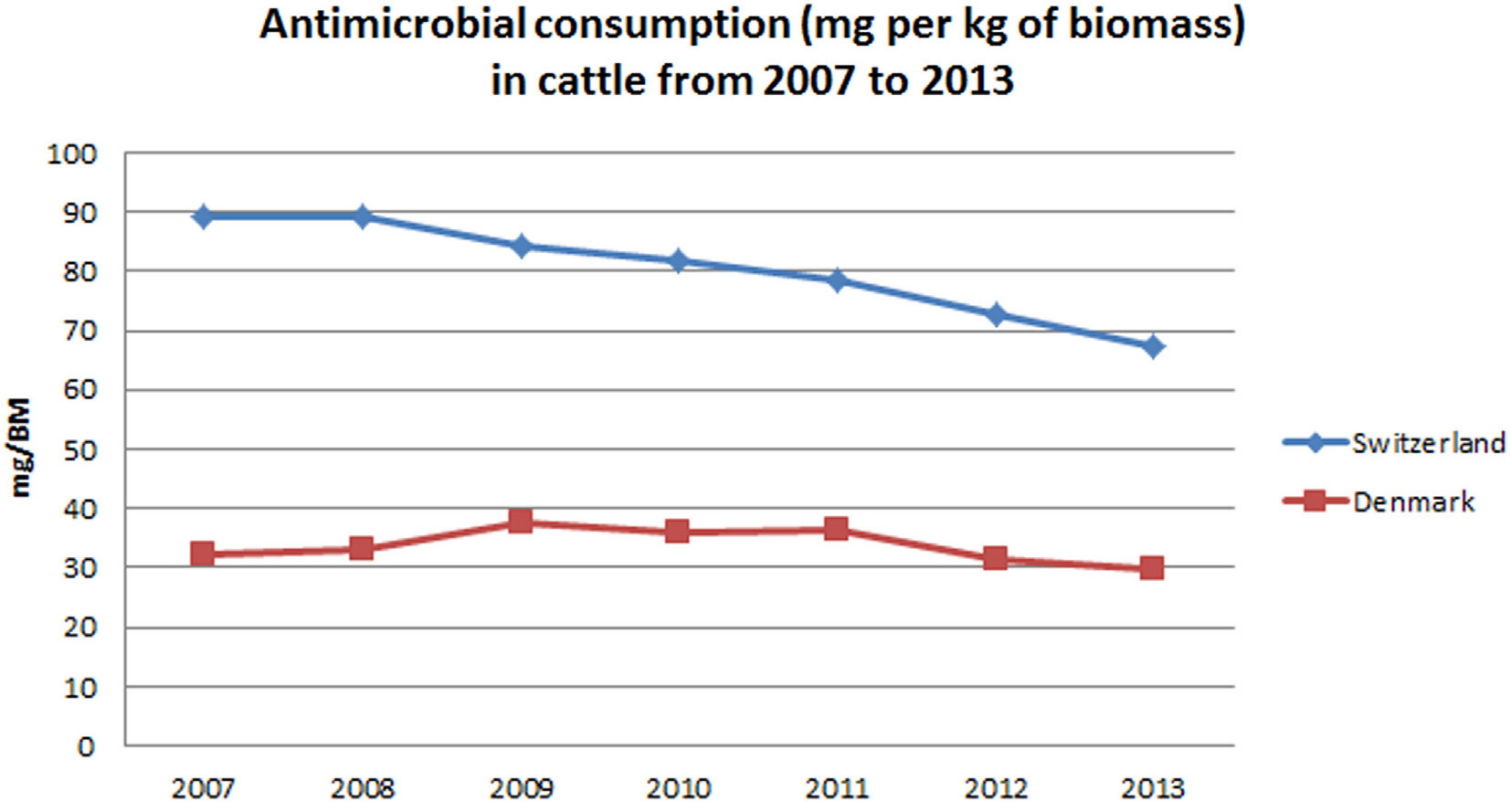
**Temporal patterns in antimicrobial consumption for cattle in Denmark and Switzerland from 2007 to 2013**.

Calculated antimicrobial consumption in the Swiss cattle population was more than twice as high as observed in Denmark. In Switzerland, a 24.5% reduction was observed from a maximum of 89.3 [51.3–113.2] mg/BM in 2008 to 67.4 [38.7–85.4] mg/BM in 2013. In Denmark, antimicrobial consumption in cattle was lower and varied over the study period between a maximum of 37.6 mg/BM in 2009 and a minimum of 29.8 mg/BM in 2013, which corresponds to a reduction of 20.8% (Figure 1).

### Antimicrobial Consumption Estimates in Pigs

**Figure 2:**
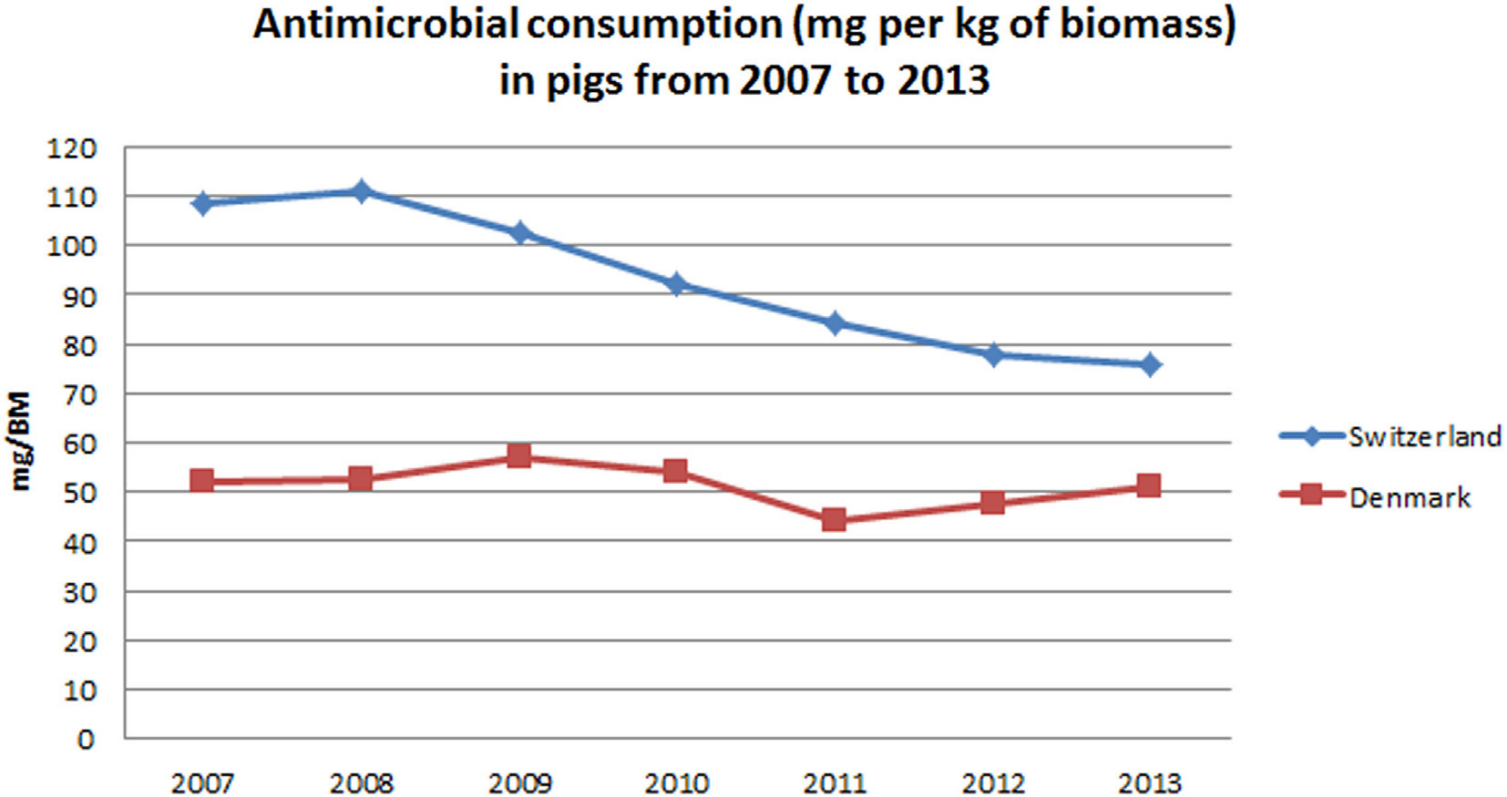
**Temporal patterns in antimicrobial consumption for pigs in Denmark and Switzerland from 2007 to 2013**.

Antimicrobial consumption for pigs in Switzerland was also higher than in Denmark. However, when compared to cattle, a steeper decrease (31.5%) of antimicrobial consumption was observed in Switzerland for pigs, from a maximum of 110.9 [54.0–201.3] mg/BM in 2007 to a minimum of 75.9 [34.0–143.8] mg/BM in 2013. In Denmark, there was a decrease of 1.9% in the consumption of antimicrobials for pigs, when comparing 2013 to 2007. It should, however, be noted that a marked reduction was achieved from 2009 (57.0 mg/BM) to 2011 (44.4 mg/BM). After this decrease, antimicrobial consumption increased again to 51.0 mg/BM in 2013 (Figure 2).

### Antimicrobial Classes’ Consumption Estimates for Cattle

The estimated antimicrobial consumption for Swiss cattle was higher for every antimicrobial class when compared to Danish cattle; except for penicillins from 2008 onward, where a lower consumption was observed for Swiss than for Danish cattle. The largest absolute differences were observed for sulfonamides/trimethoprim and tetracyclines (Table 1).

**Table 1:** **Calculated antimicrobial consumption (in milligrams per kilogram biomass) of different antimicrobial classes for cattle in Denmark and Switzerland (2007–2013)**.

Antimicrobial class	Country	2007	2008	2009	2010	2011	2012	2013	Average percentage of consumption per country
Aminoglycosides	Denmark	2.3	2.0	2.2	2.0	1.9	1.6	1.3	5.6
	Switzerland	4.3 [2.5–5.6]	4.1 [2.4–5.4]	3.8 [2.3–5.2]	3.6 [2.1–4.7]	3.7 [2.1–4.9]	3.6 [2.1–4.7]	3.4 [2.0–4.6]	4.7 [2.8–6.2]

Cephalosporins and fluoroquinolones	Denmark	0.2	0.3	0.2	0.3	0.3	0.3	0.3	0.8
	Switzerland	0.8 [0.6–1.0]	0.9 [0.6–1.1]	1.0 [0.7–1.1]	1.0 [0.8–1.2]	1.0 [0.7–1.1]	0.9 [0.7–1.0]	1.0 [0.7–1.1]	1.1 [0.9–1.3]

Macrolides	Denmark	1.1	0.8	0.8	0.5	0.6	0.5	0.5	2.0
	Switzerland	5.1 [2.3–7.2]	5.3 [2.4–7.6]	4.9 [2.2–6.9]	4.5 [2.0–6.4]	4.4 [2.0–6.2]	4.0 [1.9–5.7]	3.8 [1.7–5.3]	5.7 [2.6–8.0]

Penicillins	Denmark	17.1	19.1	22.1	22.3	22.7	20.3	19.7	60.7
	Switzerland	18.4 [12.8–22.3]	19.0 [13.0–22.9]	17.8 [12.1–21.6]	18.6 [12.4–22.8]	19.0 [12.7–23.5]	18.5 [12.5–22.6]	18.2 [12.0–22.2]	23.0 [15.5–28.0]

Sulfonamides/trimethoprim	Denmark	6.0	5.5	5.9	5.0	5.2	3.4	2.7	14.3
	Switzerland	44.5 [24.3–56.4]	42.3 [22.9–54.2]	39.8 [21.6–51.0]	37.6 [19.7–48.6]	34.3 [18.3–44.9]	31.4 [17.1–40.0]	27.5 [15.0–35.1]	45.7 [24.7–58.6]

Tetracyclines	Denmark	4.6	4.6	5.3	4.5	4.5	4.1	4.0	13.4
	Switzerland	16.1 [8.7–20.5]	17.5 [9.5–22.0]	16.7 [9.0–21.0]	16.3 [8.9–20.6]	15.8 [8.7–20.0]	14.3 [7.8–18.1]	13.3 [7.1–16.8]	19.5 [10.6–24.7]

Others	Denmark	0.9	0.9	1.1	1.1	1.4	1.3	1.3	3.3
	Switzerland	0.2 [0.1–0.3]	0.2 [0.1–0.4]	0.2 [0.1–0.4]	0.2 [0.2–0.4]	0.2 [0.2–0.4]	0.2 [0.2–0.4]	0.2 [0.2–0.4]	0.3 [0.2–0.5]

Total	Denmark	32.0	33.1	37.6	35.8	36.5	31.4	29.8	100.0
	Switzerland	89.3 [51.3–113.2]	89.3 [50.8–113.7]	84.2 [48.0–107.2]	81.9 [46.0–104.7]	78.4 [44.7–101.0]	72.9 [42.2–92.4]	67.4 [38.7–85.4]	100.0

*For Swiss estimates, the 95% credibility intervals are presented in square brackets. “Others” include amphenicoles, quinolones, lincosamides (combined or not with spectinomycin), pleuromutilins, and polypeptide antibiotics. Due to confidentiality reasons, fluoroquinolones and cephalosporins have been grouped together*.

**Figure 3:**
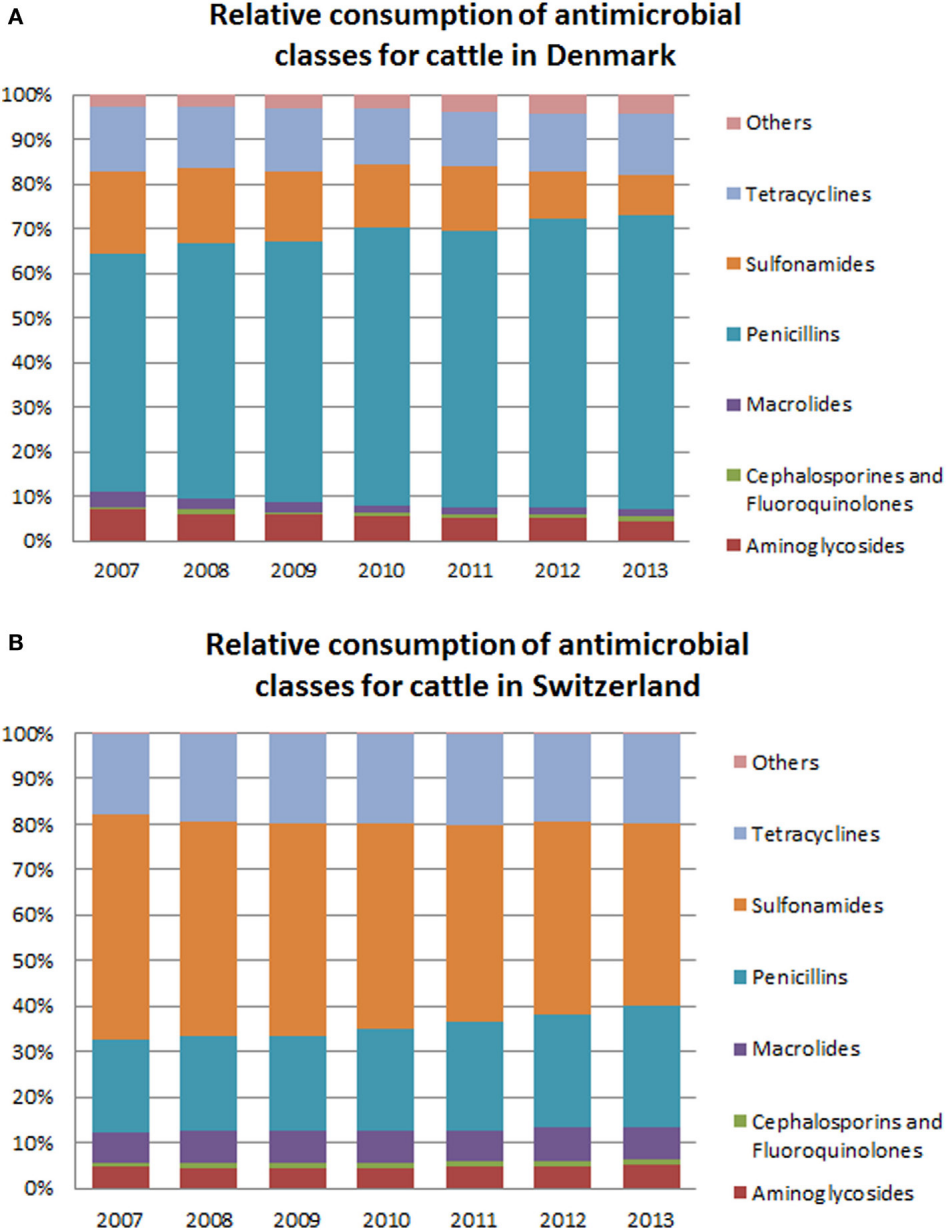
**Comparison of the relative consumption of different antimicrobial classes between Denmark (A) and Switzerland (B) for use in cattle**. “Others” include amphenicols, quinolone, lincosamide (with or not combination with spectinomycin), pleuromutilins, and polypeptide antibiotics. Due to confidentiality reasons, fluoroquinolones and cephalosporins have been grouped together.

Variations in the relative consumption of antimicrobial classes within each country are presented in Figures 3A,B. In Switzerland, sulfonamides (in combination with trimethoprim) were the antimicrobial class sold the most for cattle (between 49.8% [27.2–63.2%] of the overall mg/BM in 2007 and 40.8% [22.2–52.0%] in 2013). Simultaneously, an increase in the relative consumption of penicillins was observed. In Denmark, penicillins were the antimicrobial class with the largest consumption (between 53.3% of the total mg/BM in 2007 and 66.2% in 2013).

Important differences between the two countries were observed regarding the relative consumption of antimicrobial classes for cattle. Sulfonamides/trimethoprim, tetracyclines, macrolides, as well as cephalosporins and fluoroquinolones, had a higher relative consumption in Switzerland than in Denmark. The values for aminoglycosides were similar—between 7.0% (2007) and 4.4% (2013) for Denmark; and 4.4% [2.5–5.8%] (2010) and 5.1% [3.0–6.7%] (2013) for Switzerland. The relative consumption of penicillins in Denmark was on average 37.7% larger than in Switzerland (Table 1).

### Antimicrobial Classes’ Consumption Estimates for Pigs

Throughout the study period, the relative consumption of penicillins in swine was higher in Denmark than in Switzerland (15.9% on average—Table 2). The relative consumption of macrolides was similar for both countries. With the exception of aminoglycosides in 2007, consumption in Switzerland was higher for all other classes.

**Table 2:** **Antimicrobial consumption (in milligrams per kilogram of biomass) of different antimicrobial classes for pigs in Denmark and Switzerland (2007–2013)**.

Antimicrobial class	Country	2007	2008	2009	2010	2011	2012	2013	Average percentage of consumption per country
Aminoglycosides	Denmark	3.5	2.5	2.8	2.7	2.4	2.6	2.6	5.3
	Switzerland	3.5 [0.7–7.6]	3.6 [0.6–7.4]	3.5 [0.6–7.4]	3.1 [0.6–6.4]	3.2 [0.6–6.7]	3.0 [0.5–6.4]	3.0 [0.5–6.5]	3.5 [0.6–7.4]

Cephalosporins and fluoroquinolones	Denmark	0.1	0.1	0.1	0.0	0.0	0.0	0.0	0.1
	Switzerland	0.4 [0.1–0.9]	0.4 [0.1–1.0]	0.4 [0.1–1.0]	0.4 [0.1–0.9]	0.4 [0.1–0.9]	0.4 [0.1–0.8]	0.4 [0.1–1.0]	0.4 [0.1–1.0]

Macrolides	Denmark	6.5	6.2	7.4	7.0	5.4	6.2	6.3	12.5
	Switzerland	7.0 [2.1–13.4]	7.8 [2.3–14.8]	7.1 [2.1–13.8]	6.5 [1.8–12.7]	5.8 [1.5–11.3]	5.9 [1.8–11.2]	5.5 [1.6–10.9]	7.0 [2.0–13.5]

Penicillins	Denmark	13.4	13.1	14.7	14.7	12.8	12.9	13.7	26.6
	Switzerland	9.4 [2.4–21.1]	10.4 [2.6–23.4]	10.4 [3.0–22.6]	9.8 [1.9–23.1]	10.2 [2.0–23.5]	9.5 [1.4–22.9]	10.1 [2.0–23.9]	10.7 [2.3–24.6]

Sulfonamides/trimethoprim	Denmark	3.9	4.1	5.0	4.8	3.9	4.2	5.0	8.7
	Switzerland	41.1 [14.8–84.7]	44.5 [16.6–89.6]	41.1 [15.4–82.6]	36.9 [12.6–74.3]	31.6 [9.5–67.2]	31.7 [12.0–64.9]	28.4 [10.7–58.2]	39.1 [14.0–79.9]

Tetracyclines	Denmark	18.9	18.6	19.4	17.4	14.2	15.9	16.8	33.8
	Switzerland	40.5 [31.3–56.3]	38.2 [27.4–57.0]	33.9 [24.1–51.4]	29.7 [20.7–45.6]	26.4 [17.6–42.0]	22.5 [14.1–36.8]	23.7 [15.6–37.6]	32.9 [23.1–50.1]

Others	Denmark	5.7	7.8	7.7	7.5	5.7	5.7	6.6	13.0
	Switzerland	6.8 [5.5–8.6]	6.0 [4.4–7.9]	5.9 [4.5–7.7]	5.6 [4.5–7.2]	6.9 [5.4–8.6]	5.0 [3.9–6.3]	4.7 [3.5–5.8]	6.3 [4.9–8.0]

Total	Denmark	52.0	52.4	57.0	54.2	44.4	47.5	51.0	100.0
	Switzerland	108.6 [56.7–192.3]	110.9 [54.0–201.3]	102.4 [49.8–186.4]	92.1 [42.1–170.2]	84.4 [36.6–160.3]	78.0 [33.7–149.4]	75.9 [34.0–143.8]	100.0

*For Swiss estimates, the 95% credibility intervals are presented in square brackets. “Others” include amphenicols, quinolone, lincosamides (with or not combination with spectinomycin), pleuromutilins, and polypeptide antibiotics. Due to confidentiality reasons, fluoroquinolones and cephalosporins have been grouped together*.

As observed for cattle, the differences in antimicrobial consumption between the two countries were especially pronounced for sulfonamides/trimethoprim and tetracyclines (Table 2).

**Figure 4:**
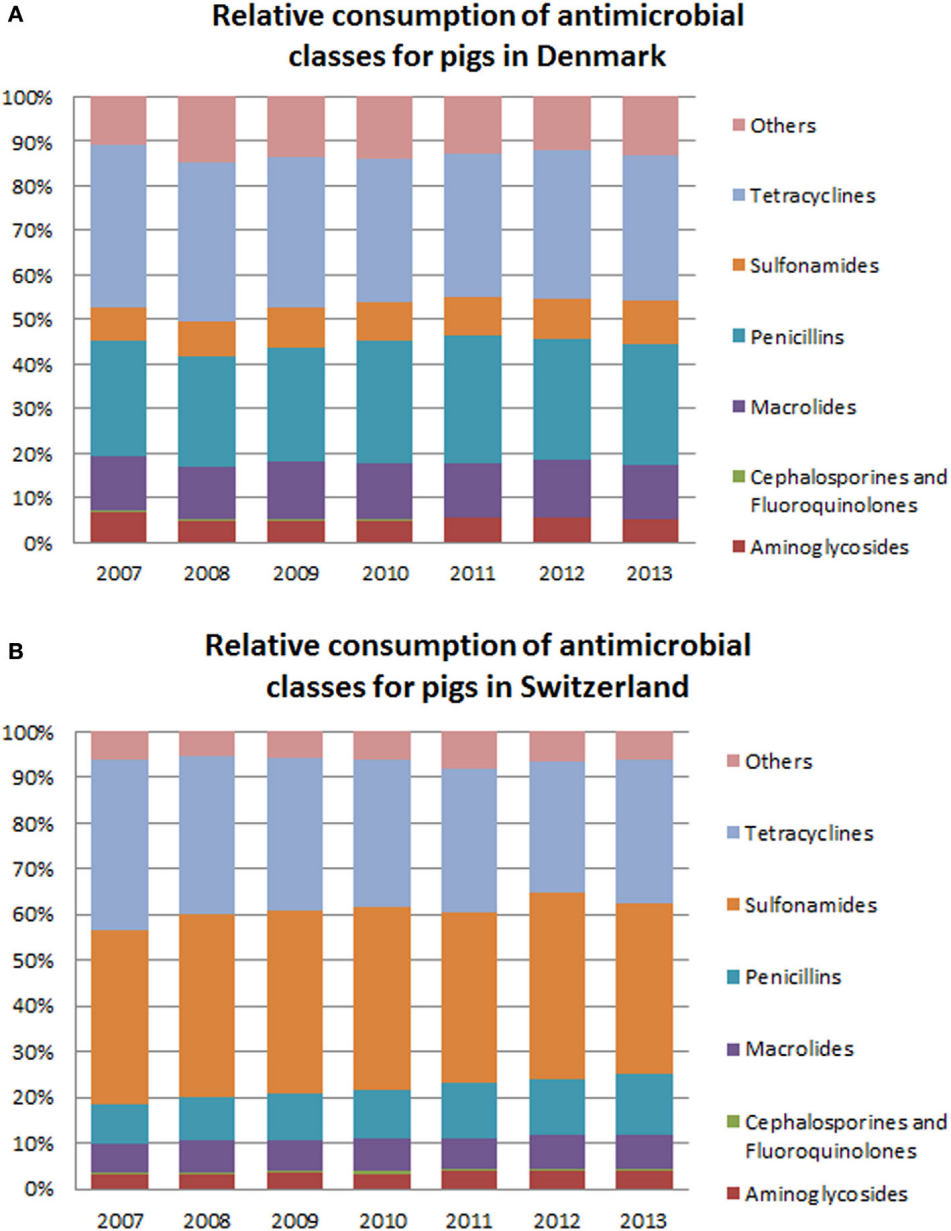
**Comparison of the relative consumption of different antimicrobial classes between Denmark (A) and Switzerland (B) for use in pigs**. “Others” include amphenicols, quinolone, lincosamide (with or not combination with spectinomycin), pleuromutilins, and polypeptide antibiotics. Due to confidentiality reasons, fluoroquinolones and cephalosporins have been grouped together.

In line with what was observed for cattle, within-country variation in the relative consumption of antimicrobial classes throughout the years was not large (Figures 4A,B). In Switzerland, there was a decrease in the relative consumption of tetracyclines, from a maximum of 37.2% [28.8–51.8%] of the total mg/BM in 2008 to a minimum of 28.8% [18.1–47.2%] in 2012. At the same time, there was an increase in the relative consumption of penicillins and macrolides. In Denmark, the proportion of antimicrobial classes used was rather stable, with only few fluctuations.

We observed clear differences between the two countries with respect to the relative consumption of antimicrobial classes for pigs. Sulfonamides/trimethoprim had a lower relative consumption in Denmark (between 7.5% of the total mg/BM in 2008 and 9.8% in 2013) when compared with Switzerland (minimum of 37.4% [14.1–76.6%] in 2013 and a maximum of 40.2% [15.4–83.2%] in 2012). On the other hand, macrolides and penicillins had a relatively higher consumption in Denmark than in Switzerland.

### Correlation Analysis

A correlation analysis was performed to assess the relationship between antimicrobial consumption and the years included in the study period. The majority of combinations of species/country/antimicrobial class presented a negative correlation coefficient (Table 3). The closer the correlation coefficients are to −1 or 1, the strongest the negative or positive, respectively, linear association between the years and antimicrobial consumption.

**Table 3:** **Correlation coefficients between years (2007–2013) and antimicrobial consumption for each combination of species/country/antimicrobial class**.

Country	Antimicrobial class	Correlation coefficient	
		Cattle	Pigs
Denmark	Aminoglycosides	−0.91	−0.61
Switzerland	Aminoglycosides	−0.94	−0.90
Denmark	Cephalosporins and fluoroquinolones	0.63	−0.87
Switzerland	Cephalosporins and fluoroquinolones	0.40	NA
Denmark	Macrolides	−0.88	−0.31
Switzerland	Macrolides	−0.97	−0.89
Denmark	Penicillins	0.41	−0.13
Switzerland	Penicillins	−0.07	0.02
Denmark	Sulfonamides/trimethoprim	−0.90	0.37
Switzerland	Sulfonamides/trimethoprim	−1.00	−0.93
Denmark	Tetracyclines	−0.62	−0.71
Switzerland	Tetracyclines	−0.83	−0.98
Denmark	Others	0.94	−0.27
Switzerland	Others	NA	−0.66
Denmark	Total antimicrobial consumption	−0.29	−0.47
Switzerland	Total antimicrobial consumption	−0.98	−0.97

*“Others” include amphenicols, quinolone, lincosamides (with or not combination with spectinomycin), pleuromutilins, and polypeptide antibiotics. Due to confidentiality reasons, fluoroquinolones and cephalosporins have been grouped together*.

## Discussion

The antimicrobial consumption in cattle and pigs varied considerably between Denmark and Switzerland in the considered study period (2007–2013). Furthermore, marked differences were found between the countries regarding each species’ relative consumption of different antimicrobial classes.

### Comparison with Other Studies

Differences in the use of antimicrobials in different animal species have already been described. Sjölund et al. found variations in the relative usage of different antimicrobial classes in farrow-to-finish pig herds from four European countries (25). Moreover, a comparative study on antimicrobial exposure between Denmark and The Netherlands detected differences in the consumption patterns of several substances in terms of routes of administration, relative consumption of antimicrobial classes, and antimicrobial consumption in different age classes (26).

Even though the amount of marketed active substance is not an ideal measure of usage, the larger amount used in Switzerland compared to Denmark very likely reflects a true difference in usage practice, both in quantities and patterns of use. These findings are in line with the general trend described in the ESVAC reports (1–5) for the same period. Nordic countries (Denmark included) are well known for their conservative use of antimicrobials and many years of activities in place to reduce antimicrobial consumption (18, 27–29). While Denmark is one of the countries participating in the ESVAC project with a lower amount of antimicrobials sold per kilogram of animal biomass, Switzerland is in the average (1–5).

### Consumption Patterns and Potential Contributing Factors

It should be stressed that this study was not conducted to assess the factors behind the observed differences between the countries, but rather to compare (at the species level) the antimicrobial consumption patterns in Denmark and Switzerland. Future investigations should provide a formal evaluation of these discrepancies. In this section, potential contributing factors are discussed based on the existing literature and on the authors’ knowledge about the veterinary practices and the animal production situation in the countries.

Both countries considered in this study presented a decrease in antimicrobial consumption (measured in mg/BM) for cattle and pigs when comparing 2013 to 2007. This is also attested by the fact that the majority of species/country/antimicrobial class combinations presented a negative correlation between years and amount of antimicrobial consumed (in mg/BM), as shown in Table 3.

Throughout this period, Denmark and Switzerland implemented several measures that contributed to the observed reduction. In Denmark, the introduction of the “Yellow Card” system in 2010 induced a remarkable drop in the consumption of antimicrobials in pigs in 2010 and 2011 (28, 30), as shown in Figure 2. The “Yellow card” is a benchmarking system for antimicrobial consumption at the farm level. It was first implemented in the pig production sector and expanded to the cattle production sector in 2011. Initially, permit limits were set at twice the average of the antimicrobial consumption in a given age group. Farmers using more than twice the average are subjected to restrictions imposed by the Danish Veterinary and Food Administration (28). Despite the subsequent increase in antimicrobial consumption in pigs from 2011 to 2013, it should be noted that a 10.0% decrease in the tonnage of prescribed antimicrobials for pigs has been observed from 2013 to 2015 (31). This followed the implementation of lower permit limits by the Danish Food and Veterinary Administration in September 2012 and again in February 2014 (32). In Switzerland, multiple factors might have contributed to the observed reduction. First, several educational initiatives presumably increased awareness among farmers and veterinarians regarding the risks associated with antimicrobial usage and the associated development of antimicrobial resistance. These educational programmes started after the introduction of the Tierarzneimittelverordnung (Ordinance on veterinary drugs) in 2004 (33) and have been gradually implemented since then. Since 2015, these various programs are coordinated within a national strategy to reduce selection and development of bacterial resistances [StAR program (34)]. Additionally, the commercialization of vaccines (against *Lawsonia intracellularis* in 2006 and Porcine Circovirus-2 in 2009) might have also played a role in the pig sector. In cattle, more specifically, the Bovine Viral Diarrhea eradication program initiated in 2008 (35) probably also contributed to the observed trend, as animals became less susceptible to secondary bacterial infections (36).

When comparing 2013 to 2007, the total reduction in the calculated antimicrobial consumption in Switzerland was 30.1% for pigs and 24.5% for cattle, while Denmark—from an already lower level—achieved a total reduction of 1.9% in pigs and 6.9% in cattle. We hypothesize that these differences are partly related to the fact the Switzerland had a much higher consumption than Denmark in the beginning of the study period. It is more difficult for a system closer to the lower limit of antimicrobial usage, such as the Danish one, to achieve large reductions, than for a system with higher antimicrobial consumption and multiple opportunities for improvement. This was already demonstrated in The Netherlands where, through a series of interventions, a reduction of 56% between 2007 and 2012 (37) was achieved. But, the overall antimicrobial consumption in The Netherlands was the highest (179 mg/PCU) among the nine countries (Switzerland and Denmark being among those) included in the first ESVAC report (1).

The results were corrected for the population size and species; therefore, differences related to these factors are not expected to have an effect on the results. Differences in animal health status could provide an obvious explanation for a higher need for antimicrobials. As Switzerland is currently free of porcine respiratory and reproductive syndrome (PRRS) and has nearly eradicated enzootic pneumonia and porcine pleuropneumonia (38), but still uses more antimicrobials compared to Denmark, where these diseases are still present (39, 40), additional factors must be sought to explain the differences in the pig sector. One explanation may be the Danish specific pathogen-free (SPF) system, which was initiated in 1971 (41). More than 70% of sow herds are part of the SPF system, and a substantial part of the finishing herds are either part of the SPF system or comply with the biosecurity rules set by the SPF system. Herds in the system are monitored for absence of the most important swine infections such as PRRS, *Mycoplasma hyopneumoniae, Pasteurella multocida, Brachyspira hyodysenteriae, Actinobacillus pleuropneumoniae*, lice, and scabies. In the case where a herd is infected with any of these pathogens, its health status is declared in the SPF system. This declaration is made public, allowing producers to purchase replacement gilts from herds having the same status as his own.

For cattle, the disease status is similar between the countries, which, again does not explain the observed differences in antimicrobial consumption. Nevertheless, interventions implemented in Denmark might elucidate some differences. Intramammary treatments account for a large proportion of antimicrobial consumption in dairy cattle (42). In Denmark, dry-cow treatment is not allowed without a bacteriological analysis of a milk sample 35 days before administering the injectors (43).

The differences between countries in the use of other substances to substitute antimicrobials, such as zinc oxide, could partly explain some of the observed differences. Other aspects that may play a role, both in the overall antimicrobial consumption, as well as in the specific substances used, are farming conditions (44–46). Postma et al. (44) concluded that a better external biosecurity in pig farms was related to a lower usage of antimicrobials. Biosecurity is not a high priority for Swiss swine farms (47). Completely closed farms are the minority, and nearly half of all farmers practice all-in all-out production (9, 47). On the contrary, biosecurity in Denmark is of high priority and regular visits to inspect external biosecurity are a prerequisite for farms wishing to be part of the SPF system. In addition, resistance patterns of animal pathogens may also be an underlying factor influencing treatment choices (25). However, the resistance situation in both countries is favorable when compared with other European countries (48, 49).

In summary, all the previously mentioned factors can potentially explain some of the observed differences between the two countries. An interesting feature that has not been thoroughly investigated relates to the influence of education and awareness on the prescription patterns of practitioners, as well as on the treatment practices of farmers. With regards to the relative consumption of antimicrobial classes, an important factor that could shed some light on the differences observed between the countries is the existence of treatment guidelines in Denmark. In Switzerland, guidelines for specific species and indications are still under development and will be published in 2017. In Denmark, these recommendations were first issued in 1996 and have been updated throughout time (50). Focus was set on prudent use as well as on the correct choice of antimicrobials for specific treatments. This might contribute to some of the observed preference, such as the use of penicillins. Another example is the Danish Order (DK) 785/2010, which recommends the use of penicillins for the treatment of mastitis (18). Any mastitis treatment with antimicrobials other than penicillins requires the previous bacterial and susceptibility testing of a milk sample. This might partially explain the discrepancies observed between the countries on the relative proportion of antimicrobial classes administered to cattle (43). For pigs, requirements from certain markets, such as the American, regarding sulfonamides’ residues might partly explain the lower relative consumption of this class in Denmark. An additional point to consider relates to differences in product availability (51, 52). The most notable examples are the limitations in the prescription of fluoroquinolones (27) and the ban on third/fourth generation cephalosporins put in place by the Danish pig industry (29).

Given the descriptive nature of this study, we can only emphasize the need to further investigate these observations and hypothesize on potential contributing factors. This is of relevance given the need to further reduce the consumption of antimicrobials in livestock species and preserve the efficacy of these substances for human use, without jeopardizing animal health, productivity, and animal welfare.

### Study Limitations

Our study has some limitations that need to be addressed. Antimicrobial consumption estimates from Switzerland were extrapolated from sales data using information from a previous field study (12). On the other hand, Danish consumption values presented on DANMAP reports were directly taken from the VetStat database. Therefore, the data on Danish antimicrobial consumption are considered to be more accurate, since they were based on prescription data (closer to the actual usage than sales data) with a national coverage. The fact that Swiss estimates rely on a field study might have introduced some bias. It should also be noted that uncertainty around some of the Swiss estimates was large. This relates to the minimum and maximum values used in the Pert distributions. These values were calculated based on sales data (from the Swiss Federal Food Safety and Veterinary Office) as the minimum and maximum amounts of each antimicrobial class that could have been sold for consumption in a given species. For antimicrobial classes where the interval between the minimum and the maximum was larger, uncertainty was also increased. Another study limitation relates to the fact that antimicrobial consumption by dairy cattle and beef cattle were put together, for both Denmark and Switzerland. These production systems are associated with different health issues, which might lead to the use of dissimilar antimicrobial substances. Furthermore, the proportion of dairy and non-dairy herds varies considerably between the two countries: 18% dairy versus 82% non-dairy in Denmark (22) and 57% dairy versus 43% non-dairy in Switzerland (23). Nevertheless, it should be noted that the average number of animals might differ between countries and production systems. Therefore, the proportion of animals at risk of being treated (dairy versus non-dairy) with antimicrobials can be different than the percentages indicated above.

Animal age categories and types of production were not taken into account in our analysis as the Swiss antimicrobial consumption estimates were not detailed enough. However, it is known that these parameters could also influence antimicrobial consumption (25, 53) and should, if possible, be considered in future comparative studies.

### Final Remarks

In conclusion, we found substantial variations between Denmark and Switzerland on the relative consumption of different antimicrobial classes in cattle and pigs. Several factors other than animal demographics might contribute to the differences observed on overall antimicrobial sales, as well as the relative consumption of antimicrobial classes, across Europe. These factors should be further investigated to better understand drivers of antimicrobial usage and prioritize more efficient mitigation strategies.

## Author Contributions

LPC analyzed the data, interpreted the results, and wrote the manuscript. LN and LA provided valuable expertise on the topic, especially on the interpretation of Danish data. CM and GS-R also provided valuable expertise on the topic, with special emphasis on the interpretation of Swiss data. IM was the main supervisor of the PhD student and assisted the first author (LPC) in all steps of the study. All the authors actively contributed to the conception of the project, have read, and approved the manuscript.

## Conflict of Interest Statement

The authors declare that the research was conducted in the absence of any commercial or financial relationships that could be construed as a potential conflict of interest.
